# Association between early fluid overload and mortality in critically-ill mechanically ventilated children: a single-center retrospective cohort study

**DOI:** 10.1186/s12887-021-02949-w

**Published:** 2021-10-26

**Authors:** Xiangmei Kong, Yueniu Zhu, Xiaodong Zhu

**Affiliations:** Department of Pediatric Intensive Care Unit, Xinhua Hospital Affiliated to Shanghai Jiaotong University School of Medicine, Shanghai, 200092 China

**Keywords:** Fluid overload, Mortality, Mechanical ventilation, Children

## Abstract

**Background:**

Positive fluid overload (FO) may cause adverse effect. This study retrospectively analyzed the relationship between early FO and in-hospital mortality in children with mechanical ventilation (MV) in pediatric intensive care unit (PICU).

**Methods:**

This study retrospectively enrolled 309 children (ages 28 days to 16 years) receiving invasive MV admitted to the PICU of Xinhua Hospital from March 2014 to March 2019. Children receiving MV for less than 48 h were excluded. The FO in the first 3 days of MV was considered to the early FO. Patients were divided into groups according to early FO and survival to evaluate the associations of early FO, percentage FO(%FO) > 10%, and %FO > 20% with in-hospital mortality.

**Results:**

A total of 309 patients were included. The mean early FO was 8.83 ± 8.81%, and the mortality in hospital was 26.2% (81/309). There were no significant differences in mortality among different FO groups (*P* = 0.053) or in early FO between survivors and non-survivors (*P* = 0.992). Regression analysis demonstrated that use of more vasoactive drugs, the presence of multiple organ dysfunction syndrome, longer duration of MV, and a non-operative reason for PICU admission were related to increased mortality (*P* < 0.05). Although early FO and %FO > 10% were not associated with in-hospital mortality (β = 0.030, *P* = 0.090, 95% CI = 0.995–1.067; β = 0.479, *P* = 0.153, 95% CI = 0.837–3.117), %FO > 20% was positively correlated with mortality (β = 1.057, OR = 2.878, *P* = 0.029, 95% CI = 1.116–7.418).

**Conclusions:**

The correlation between early FO and mortality was affected by interventions and the severity of the disease, but %FO > 20% was an independent risk factor for in-hospital mortality in critically ill MV-treated children.

## Background

Proper fluid management is an important treatment method in critical illness to maintain good circulation capacity and tissue perfusion. Studies have confirmed the adverse effects of high levels of fluid accumulation, including deterioration of lung function, prolonged duration of mechanical ventilation (MV), and length of stay (LOS) in hospital and pediatric intensive care unit (PICU). These studies have mostly focused on pediatric acute respiratory distress syndrome (ARDS) or acute lung injury (ALI), septic shock, and the use of continuous renal replacement therapy (CRRT) [1–8]. However, there have been few studies on the treatment of critical illnesses in PICU in general [9–11]. Although the relationship between fluid overload (FO) and mortality reported by these studies is controversial, FO may be a predictor of death in critically ill children.

Research on fluid accumulation has increasingly focused on early FO. Most studies evaluate early FO as the ratio (expressed as a percentage) of the cumulative amount of fluid intake and output to weight on admission to hospital or PICU. In a retrospective study of 638 hospitalized patients receiving MV in the PICU [11], FO within 48 h of admission was not related to mortality, but it was related to deterioration of oxygenation index and prolonged MV duration in surviving patients, especially when the percentage of FO (%FO) was greater than or equal to 15%. Sutherland et al. [7] divided patients into two groups based on %FO (< 20% and ≥ 20%) for correlation analysis. The results showed that the mortality of children with %FO ≥ 20% was about 8.5 times that of children in the low %FO group. Some studies directly defined early FO as %FO ≥ 10% and also confirmed that %FO ≥ 10% often has adverse clinical consequences [6, 9, 12].

The correlation between prognosis such as mortality and early FO in children with severe illness is controversial, and there are few relevant studies compared with adults. We hypothesized that there may be a correlation between early FO and mortality in patients with severe pediatric mechanical ventilation. So in this study, we explored the associations of early FO with in-hospital mortality in children with invasive MV in PICU.

## Methods

### Patient population and study design

**Fig. 1:**
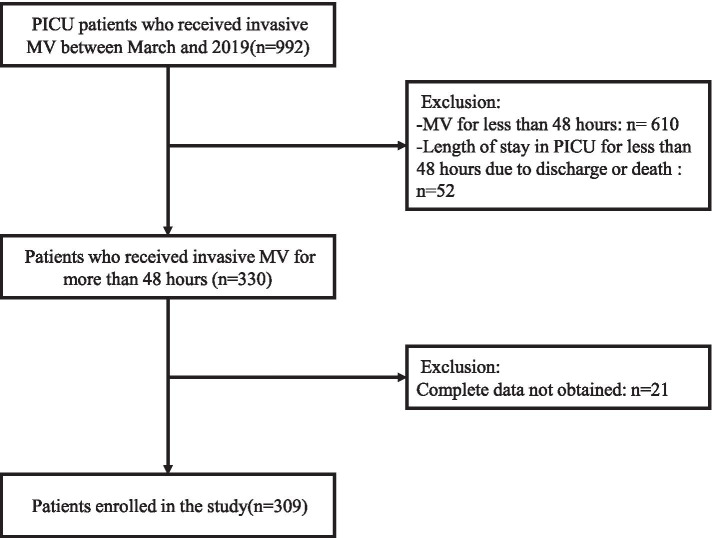
Study selection diagram. MV: mechanical ventilation. PICU: pediatric intensive care unit

This was a retrospective single center cohort study around children (aged between ≥28 days and 16 years) undergoing invasive MV admitted to the PICU of Xinhua Hospital Affiliated to Shanghai Jiaotong University School of Medicine from March 2014 to March 2019. All patients were included with MV for more than 48 h. Children who received MV for less than 48 h or were hospitalized in PICU for less than 48 h due to discharge or death were not included in this cohort (Fig. 1). Local research ethics approval for the study was obtained from the ethics committee of Xinhua Hospital Affiliated to Shanghai Jiao Tong University School of Medicine (approval no. XHEC-D-2020-163). The actual number of patients enrolled far exceeds the estimated sample size.

Daily fluid assessment was performed in all patients. Daily fluid intake included all intravenous fluid and oral rehydration; daily fluid output included urine volume, feces, all drainage volume, and continuous renal replacement therapy (CRRT), dehydration. The ratio of the difference between daily fluid intake and output to baseline weight at admission to PICU was expressed as a percentage: %FO = (daily fluid intake in liters − daily fluid output in liters)/admission weight in kilograms * 100%. We defined early FO as %FO during the first 3 days of MV. We focused on %FO > 10% and %FO > 20% and divided patients into four groups based on %FO as follows: %FO ≤ 0%, 0 < %FO ≤ 10, 10% < %FO ≤ 20%, and %FO > 20%. We also divided the children into survival group and non-survival group to explore whether there was a difference in early FO.

### Data collection and definition

Basic demographic information was collected, including: age, gender, baseline weight at admission to PICU, presence of underlying disease, duration of MV, LOS in hospital, LOS in PICU, presence of multiple organ dysfunction syndrome (MODS), in-hospital mortality, main intervention measures (receipt of CRRT, use of vasoactive drugs), and daily fluid access.

Patients were grouped based on their main reason for PICU admission into surgical patients and medical patients. The third-generation admission pediatric risk of mortality score (PRISM-III) was used as the measure of illness severity and was determined for all patients during the first 24 h following admission to the PICU. Vasoactive medications were defined as any continuous vasoactive infusion used for cardiovascular support. Based on any chronic condition on admission, underlying disease mainly included congenital malformation, immune deficiency, genetic and metabolic diseases, benign and malignant tumors, and severe malnutrition that were present and clearly diagnosed before admission. MODS was defined as at least two failed organs at any time during PICU admission, according to recently published criteria [13]. Clinical outcomes measured included length of PICU stay, LOS in hospital, and in-hospital mortality that was defined as a death occurring during hospital stay. The duration of MV in days was measured as the time from first MV support to extubation or time of PICU discharge without extubation. Extubation failure was defined as the reinstitution of MV within 48 h of extubation. Duration of MV longer than 7 days was concerned based on the effect of prolonged MV on prognosis [14, 15].

### Clinical outcomes

The main purpose of this study was to investigate the relationships of early FO, %FO > 10%, and %FO > 20% with in-hospital mortality. The secondary objective was to study the relationships of early FO with LOS in hospital and LOS in the PICU.

### Statistical analysis

SPSS Statistics version 22.0 (IBM, Armonk, NY) was used for statistical analysis. Kruskal–Wallis test was used to analyze continuous variables in different groups, and Mann–Whitney U-test was used to analyze continuous variables between survivors and non-survivors. Pearson chi-square test was used for categorical variables. For continuous variables, data were reported as median with interquartile range (IQR) or mean ± standard deviation (SD); percentages were used for categorical variables. The relationship between early FO and LOS in PICU or hospital was assessed based on the Spearman rank correlation coefficient. A binary multivariate logistic regression model was used to analyze the effects on in-hospital mortality. Other outcome measures with *P* < 0.1 were introduced in the multivariate logistic regression model. Results were presented as odds ratios (ORs) with 95% confidence intervals (CIs) for logistic regression. *P*-value less than 0.05 was considered significant.

## Results

### Demographics of all subjects

We collected and analyzed cases from the past 5 years; 309 patients were eligible for inclusion. The characteristics of the patients were shown in Table 1. There were 107 cases in the operative group and 202 cases in the non-operative group. The in-hospital mortality was 26.2% (81/309), 187 patients were male (60.5%), and more than half of the patients (69.3%) had underlying diseases. 59 patients (19.1%) received CRRT, 91 patients (29.4%) were diagnosed with MODS, and the median PRISM-III score was 5.0.

**Table 1:** Patient characteristics

Variable	***N*** = 309
**Age (months, m), median (IQR)**	**13.4 (4.7–52.6)**
**Weight (kg)**	**8.8 (6.0–15.0)**
**Gender, N (%)**	
**Male**	**187 (60.5%)**
**Female**	**122 (39.5%)**
**Underlying disease, N (%)**	**214 (69.3%)**
**Reason for PICU admission, N (%)**	
**Medical, N (%)**	**202 (65.4%)**
**Surgical, N (%)**	**107 (34.6%)**
**Receipt of CRRT, N (%)**	**59 (19.1%)**
**Receipt of vasoactive drugs, N (%)**	**158 (51.1%)**
**No. of vasoactive drugs, median (IQR)**	**1.0 (0.0–2.0)**
**Diagnosis of MODS, N (%)**	**91 (29.4%)**
**PRISM-III scores, median (IQR)**	**5.0 (2.0–9.0)**
**In-hospital mortality, N (%)**	**81 (26.2%)**
**Time from hospital admission to PICU (days), median (IQR)**	**0.0 (0.0–5.0)**
**LOS in PICU (days), median (IQR)**	**18.0 (9.0–27.5)**
**LOS in hospital (days), median (IQR)**	**27.0 (17.0–43.0)**
**Time of MV, median (IQR)**	**1 (1-1)**
**Duration of MV (days), median (IQR)**	**6.0 (4.0–12.0)**
**Duration of first MV (days), median (IQR)**	**6.0 (4.0–11.0)**
**Proportion of patients receiving MV for more than 7 days, N (%)**	**124 (40.1%)**
**Early FO, mean ± SD**	**8.38 ± 8.81**
**Percentage of %FO > 10%, N (%)**	**131 (42.4%)**
**Percentage of %FO > 20%, N (%)**	**27 (8.7%)**

LOS: length of stay; MV: mechanical ventilation; MODS: multiple organ dysfunction syndrome; CRRT: continuous renal replacement therapy; PRISM-III: third-generation admission pediatric risk of mortality score; PICU: pediatric intensive care unit; FO: fluid overload. No.: number. Continuous variables are reported as median (interquartile range, IQR) or mean ± SD

### Characteristics of early FO

**Fig. 2:**
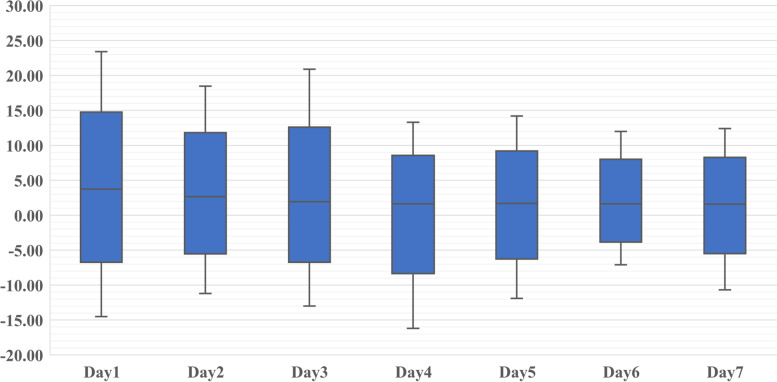
The median value of daily FO in the first 7 days

The mean early FO was 8.83 ± 8.81%. And 42.4 and 8.7% of patients had a FO of more than 10 and 20% (Table 1). The median value of daily FO was gradually stabilized (Fig. 2). The proportion of four different FO was shown in Table 2, of which 0-10% and 10-20% were more. Different early FO levels were correlated with age, weight, presence of underlying diseases, reason for PICU admission, use of vasoactive drugs and CRRT, presence of MODS, and PRISM-III scores. There was no statistical difference in duration of MV. As shown in Table 3, the median values of eraly FO in survivors and non-survivors were 8.1 and 7.6%. More patients in the death group had a FO of more than 20%(*P* = 0.037).

**Table 2:** Comparison of all patients by %FO group

Variable	%FO ≤ 0%	0% < %FO ≤ 10%	10% < %FO ≤ 20%	%FO>20%	P-value
**Number, N (%)**	**53 (17.2%)**	**125 (40.5%)**	**104 (33.6%)**	**27 (8.7%)**	
**Age (months, m), median (IQR)**	**42.4 (14.9–88.3)**	**18.5 (4.2–60.4)**^**a,c**^	**7.5 (4.4–19.9)**^**a**^	**5.8 (3.5–14.8)**^**a**^	**<0.001**
**Weight (kg)**	**15.0 (9.5–25.0)**	**11.0 (6.6–19.5)**	**7.0 (5.4–11.0)**^**a,b**^	**5.8 (4.9–8.2)**^**a,b**^	**<0.001**
**Gender, N (%)**					**0.747**
**Male**	**29 (54.7%)**	**77 (61.6%)**	**63 (60.6%)**	**18 (66.7%)**	
**Female**	**24 (45.3%)**	**48 (39.4%)**	**41 (39.4%)**	**9 (33.3%)**	
**Underlying disease, N (%)**	**26 (49.1%)**	**81 (64.8%)**	**84 (80.8%)**^**a,b**^	**23 (85.2%)**^**a,b**^	**<0.001**
**Reason for PICU admission, N (%)**					**0.030**
**Medical, N (%)**	**39 (73.6%)**	**85 (68.0%)**	**57 (54.8%)**^**a,b,d**^	**21 (77.8%)**	
**Surgical, N (%)**	**14 (26.4%)**	**40 (32.0%)**	**47 (45.2%)**^**a,b,d**^	**6 (22.2%)**	
**Receipt of CRRT, N (%)**	**22 (41.5%)**	**25 (20.0%)**^**a**^	**7 (6.7%)**^**a,b**^	**5 (18.5%)**^**a**^	**<0.001**
**Receipt of vasoactive drugs, N (%)**	**38 (71.7%)**	**62 (49.6%)**^**a**^	**41 (39.4%)**^**a,d**^	**17 (63.0%)**	**0.001**
**No. of vasoactive drugs, median (IQR)**	**2.0 (0.0–3.0)**^**b,c**^	**1.0 (0.0–2.0)**	**0.0 (0.0-1.0)**	**1.0 (0.0–2.0)**	**<0.001**
**Diagnosis of MODS, N (%)**	**28 (52.8%)**^**b,c**^	**35 (28.0%)**	**19 (18.3%)**	**9 (33.3%)**	**0.001**
**PRISM-III scores, median (IQR)**	**8.0 (5.5–12.5)**^**b,c**^	**4.0 (2.5–8.0)**	**4.0 (1.0–8.0)**	**5.0 (2.0–10.0)**	**0.001**
**In-hospital mortality, N (%)**	**17 (32.1%)**	**31 (24.8%)**	**21 (20.2%)**	**12 (44.4%)**	**0.053**
**Time from hospital admission to PICU (days), median (IQR)**	**0.0 (0.0–5.0)**	**0.0 (0.0–4.5)**	**1.0 (0.0–5.0)**	**0.0 (0.0–4.0)**	**0.356**
**LOS in PICU (days), median (IQR)**	**16.0 (9.0–22.0)**	**16.0 (9.0–27.0)**	**18.5 (10.0–31.0)**	**21.0 (9.0–32.0)**	**0.240**
**LOS in hospital (days), median (IQR)**	**27.0 (18.5–42.0)**	**25.0 (16.0–42.0)**	**30.0 (18.0–46.8)**	**27.0 (15.0–44.0)**	**0.240**
**Time of MV, median (IQR)**	**1 (1–1)**	**1 (1–1)**	**1 (1–1)**	**1 (1–1)**	**0.780**
**Duration of MV (days), median (IQR)**	**5.0 (4.0–14.0)**	**6.0 (4.0–11.0)**	**7.0 (4.0–13.8)**	**7.0 (5.0–14.0)**	**0.733**
**Duration of first MV (days), median (IQR)**	**5.0 (4.0–13.0)**	**6.0 (4.0–10.0)**	**7.0 (4.0–12.0)**	**7.0 (5.0–14.0)**	**0.625**
**Proportion of patients receiving MV for more than 7 days, N (%)**	**20 (37.7%)**	**46 (36.8%)**	**47 (45.2%)**	**11 (10.8%)**	**0.616**
**Early FO, median (IQR)**	**−3.7 (−6.9 to − 1.2)**	**5.8 (3.0–7.7)**	**14.2 (12.0–16.9)**	**23.1 (21.0–24.3)**	

LOS: length of stay; MV: mechanical ventilation; MODS: multiple organ dysfunction syndrome; CRRT: continuous renal replacement therapy; PRISM-III: third-generation admission pediatric risk of mortality score; PICU: pediatric intensive care unit; FO: fluid overload. No.: number. a: vs. %FO ≤ 0%; b: vs. 0%<%FO ≤ 10%; c: 10% < %FO ≤ 20%; d: vs. %FO > 20%. Continuous variables: *P*-values obtained by Kruskal–Wallis test; categorical variables: P-values obtained by Pearson chi-square test

**Table 3:** Comparison of survivors with non-survivors

Variable	Survivors	Non-survivors	P-value
**Number, N (%)**	**228 (73.8%)**	**81 (26.2%)**	
**Age (months, m), median (IQR)**	**11.5 (4.4–48.6)**	**16.4 (5.1–56.6)**	**0.264**
**Weight (kg)**	**8.6 (5.8–15.0)**	**10.0 (6.0–15.0)**	**0.440**
**Gender, N (%)**			**0.692**
**Male**	**136 (59.6%)**	**51 (63.0%)**	
**Female**	**92 (40.4%)**	**30 (37.0%)**	
**Underlying disease, N (%)**	**155 (68.0%)**	**59 (72.8%)**	**0.484**
**Reason for PICU admission, N (%)**			**<0.001**
**Medical, N (%)**	**135 (59.2%)**	**67 (82.7%)**	
**Surgical, N (%)**	**93 (40.8%)**	**14 (17.3%)**	
**Receipt of CRRT, N (%)**	**33 (14.5%)**	**26 (32.1%)**	**0.001**
**Receipt of vasoactive drugs, N (%)**	**96 (42.1%)**	**62 (76.5%)**	**<0.001**
**No. of vasoactive drugs, median (IQR)**	**0.0 (0.0–1.0)**	**2.0 (1.0–3.0)**	**<0.001**
**Diagnosis of MODS, N (%)**	**37 (16.2%)**	**54 (66.7%)**	**<0.001**
**PRISM-III score, median (IQR)**	**5.0 (2.3–9.0)**	**6.0 (2.0–12.5)**	**0.098**
**Time from hospital admission to PICU (days), median (IQR)**	**0.0 (0.0–5.0)**	**0.0 (0.0–3.5)**	**0.153**
**LOS in PICU (days), median (IQR)**	**18.0 (11.0–27.8)**	**16.0 (6.5–28.0)**	**0.088**
**LOS in hospital (days), median (IQR)**	**30.0 (20.0–46.8)**	**17.0 (8.0–31.5)**	**<0.001**
**Time of MV, median (IQR)**	**1.0 (1.0–1.0)**	**1.0 (1.0–1.0)**	**0.064**
**Duration of MV (days), median (IQR)**	**6.0 (4.0–11.0)**	**9.0 (5.0–22.0)**	**0.001**
**Duration of first MV (days), median (IQR)**	**6.0 (4.0–10.0)**	**9.0 (5.0–19.0)**	**0.001**
**Proportion of patients receiving MV for more than 7 days, N (%)**	**77 (33.8%)**	**47 (58.0%)**	**<0.001**
**Early FO, median (IQR)**	**8.1 (1.5–13.9)**	**7.6 (1.7–16.1)**	**0.992**
**Percentage of %FO > 10%, N (%)**	**98 (43.0%)**	**33 (40.7%)**	**0.794**
**Percentage of %FO > 20%, N (%)**	**15 (6.6%)**	**12 (14.8%)**	**0.037**

LOS: length of stay; MV: mechanical ventilation; MODS: multiple organ dysfunction syndrome; CRRT: continuous renal replacement therapy; PRISM-III: third-generation admission pediatric risk of mortality score; PICU: pediatric intensive care unit; FO: fluid overload. No.: number. Continuous variables: *P*-values obtained by Mann–Whitney U-test; categorical variables: P-values obtained by Pearson chi-square test

Compared with the other groups, the %FO ≤ 0% group was older and had more CRRT treatment; patients also received more vasoactive drugs, were more likely to have MODS, and had higher PRISM-III scores than the 0% < %FO ≤ 10 and 10% < FO ≤ 20% groups. They were heavier than those in the 10% < %FO ≤ 20% and %FO > 20% groups. Compared with the 10% < %FO ≤ 20% group, patients in the 0% < %FO ≤ 10% group were older and received more CRRT treatment; they were also heavier than those in the 10% < FO ≤ 20% and %FO > 20% groups. Compared with the other groups, the 10% < %FO ≤ 20% group had fewer medical patients and more surgical patients. Compared with the %FO ≤ 0 and 0% < %FO ≤ 10% groups, patients in the 10% < %FO ≤ 20% and %FO > 20% groups had more underlying diseases. The %FO > 20% group had more use of vasoactive drugs than the 10% < FO ≤ 20% group (all *P* < 0.05) (Table 2).

### Association between early FO and mortality

In the study, there were 228 survivors and 81 non-survivors. As shown in Table 2, there were no significant difference in the in-hospital mortality between four early FO groups(*P* = 0.053), but the group with %FO>20% had the highest mortality. As shown in Table 3, there was no statistical difference between survivors and non-survivors in early FO(*P* = 0.992); However, there were statistically significant differences in duration of MV, LOS in hospital, use of vasoactive drugs and CRRT, presence of MODS, and operative reason for admission (all *P* < 0.05). Compared with the survivors, the non-survivor group included more medical patients, received more vasoactive drugs and CRRT, and had a longer duration of MV and shorter LOS in hospital.

On multivariate analysis, the duration of MV overlapped with the duration of first MV, so we chose the duration of MV as the one of intervention factors. We adjusted for prespecified variables (number of vasoactive drugs, time of MV, duration of MV, CRRT, diagnosis of MODS, reason for PICU admission, and PRISM-III score) to evaluate the association between early FO/%FO > 10% /%FO > 20% and in-hospital mortality (Tables 4, 5, 6). The results showed that use of more vasoactive drugs, the presence of MODS, a longer duration of MV, and non-operative reason for PICU admission were related to increased mortality (all *P* < 0.05). Although it was not statistically significant, there was a positive correlation between early FO and mortality (β = 0.030, *P* = 0.090, 95% CI = 0.995–1.067) (Table 4). Similar results were obtained with logistic regression for %FO > 10% and mortality. There was no statistical correlation between %FO > 10% and mortality (β = 0.479, *P* = 0.153, 95% CI = 0.837–3.117) (Table 5), but %FO > 20% was related to increased mortality (β = 1.057, OR = 2.878, *P* = 0.029, 95% CI = 1.116–7.418) (Table 6).

**Table 4:** Multivariate log regression analysis for association of early FO with in-hospital mortality

Outcome measure	β	OR	95% C.I.	P-value
**No. of vasoactive drugs**	**0.413**	**1.511**	**1.107–2.063**	**0.009**
**Time of MV**	**−0.080**	**0.923**	**0.428–1.991**	**0.838**
**Duration of MV**	**0.045**	**1.046**	**1.017–1.075**	**0.001**
**Receipt of CRRT**	**−0.329**	**0.720**	**0.316–1.638**	**0.433**
**Diagnosis of MODS**	**1.724**	**5.609**	**2.570–12.239**	**<0.001**
**Surgery**	**−0.869**	**0.419**	**0.190–0.924**	**0.031**
**Early FO**	**0.030**	**1.031**	**0.995–1.067**	**0.090**
**PRISM-III score**	**0.016**	**1.016**	**0.964–1.071**	**0.561**

Hosmer–Lemeshow *P* = 0.805; predicted percentage 80.3%. OR: odds ratio; 95% CI: 95% confidence interval; MV: mechanical ventilation; MODS: multiple organ dysfunction syndrome; CRRT: continuous renal replacement therapy; PRISM-III: third-generation admission pediatric risk of mortality score; FO: fluid overload. No.: number

**Table 5:** Multivariate log regression analysis for association of %FO > 10% with in-hospital mortality

Outcome measure	β	OR	95% C.I.	P-value
**No. of vasoactive drugs**	**0.412**	**1.510**	**1.106–2.063**	**0.010**
**Time of MV**	**−0.043**	**0.958**	**0.444–2.065**	**0.913**
**Duration of MV**	**0.044**	**1.045**	**1.017–1.074**	**0.002**
**Receipt of CRRT**	**−0.343**	**0.710**	**0.314–1.603**	**0.409**
**Diagnosis of MODS**	**1.718**	**5.572**	**2.563–12.115**	**<0.001**
**Surgery**	**−0.886**	**0.412**	**0.188–0.904**	**0.027**
**FO > 10%**	**0.479**	**1.615**	**0.837–3.117**	**0.153**
**PRISM-III score**	**0.011**	**1.011**	**0.960–1.065**	**0.684**

Hosmer–Lemeshow *P* = 0.894; predicted percentage 79.6%. OR: odds ratio; 95%C.I.: 95% confidence interval; MV: mechanical ventilation; MODS: multiple organ dysfunction syndrome; CRRT: continuous renal replacement therapy; PRISM-III: third-generation admission pediatric risk of mortality score; FO: fluid overload. No.: number

**Table 6:** Multivariate log regression analysis for association of %FO > 20% with in-hospital mortality

Outcome measure	β	OR	95% C.I.	P-value
**No. of vasoactive drugs**	**0.385**	**1.469**	**1.079–2.000**	**0.014**
**Time of MV**	**−0.039**	**0.962**	**0.442–2.091**	**0.922**
**Duration of MV**	**0.046**	**1.047**	**1.018–1.076**	**0.001**
**Receipt of CRRT**	**−0.431**	**0.650**	**0.287–1.470**	**0.301**
**Diagnosis of MODS**	**1.733**	**5.656**	**2.591–12.345**	**< 0.001**
**Surgery**	**−0.886**	**0.412**	**0.185–0.916**	**0.030**
**FO > 20%**	**1.057**	**2.878**	**1.116–7.418**	**0.029**
**PRISM-III score**	**0.012**	**1.012**	**0.961–1.066**	**0.652**

Hosmer–Lemeshow *P* = 0.625; predicted percentage was 80.9%. OR: odds ratio; 95%C.I.: 95% confidence interval; MV: mechanical ventilation; MODS: multiple organ dysfunction syndrome; CRRT: continuous renal replacement therapy; PRISM-III: third-generation admission pediatric risk of mortality score; FO: fluid overload. No.: number

### Relationships of early FO with LOS in PICU and hospital

As shown in Table 2, there were no statistical difference in LOS in hospital and in PICU(*P* = 0.240), but %FO>20% group had longest LOS in PICU(27.0(15.0-44.0)days) and 10% < FO ≤ 20% group had longest LOS in hospital(30.0(18.0-46.8) days).

The relationships of early FO with LOS in PICU and LOS in hospital were analyzed by Spearman’s method. Although there was no significant correlation between early FO and LOS in hospital (r = 0.056, *P* = 0.33), there was a positive but weak correlation between early FO and LOS in PICU (r = 0.148, *P* = 0.009).

## Discussion

This was a retrospective study of the relationship between early FO and in-hospital mortality during invasive MV in children with critical illness. We mainly analysed the associations of early FO and in-hospital mortality adjusting for prespecified variables. The adverse effects of positive fluid accumulation have been confirmed in research on adults [1–4]. Studies of ARDS fluid management strategies have directly confirmed that compared with a positive fluid management strategy, conservative fluid treatment better achieves a negative balance of fluid management, improves lung function, and shortens LOS in ICU. Despite differences between adults and children, similar negative effects of early FO have been confirmed in studies of critical illness in children, especially in cases of ARDS/ALI, sepsis, shock, acute kidney injury (AKI), CRRT, and perioperative FO in congenital heart disease [5, 9, 16–19]. However, there have few studies of FO in multisystem diseases. These studies of the adverse effects of positive FO always exclude children with hemodynamic instability or CRRT [20]. In this study, we did not select a single disease but enrolled critically ill patients receiving MV in the PICU, including those patients with hemodynamic instability and undergoing CRRT.

In this study, early FO was defined as the accumulated FO in the first 3 days since the first day of MV. Flori et al. [16] and Valentine et al. [21] also found that the increase in FO mainly occurred in the first 3 days. Related studies on septic shock have confirmed an increase in FO in the first 72 h and its possible negative effects [6]; A study on early fluid accumulation in children with shock also showed that the peak of fluid accumulation occurred within 3 days after admission to ICU [5]. In children with severe respiratory failure who need extracorporeal life support and CRRT, it has been reported that FO occurs more in the first 24 h of fluid treatment [22]. Therefore, it is important to choose an appropriate time for early FO, in order to accurately explore the correlation between FO and prognostic factors.

In our study, the mean early FO was consistent with those of studies by Arikan et al., in which 75% of the FO in the first 2 days was 11% [20], and Valentine et al., in which the average FO in the first 3 days was 8.5 ± 10.5% [21]. Although fluid management has become one of the most important treatment measures for critically ill children, the presence of positive FO remains very common. A previous study in North America and European countries showed that only 29% of ALI patients received restrictive fluid management in clinical treatment [23]. Some studies have found that the amount of FO in children with ALI was similar in adults with positive fluid management strategy, even though a restrictive fluid therapy strategy was used [21].

The relationship between early FO and mortality has been a research hotspot. In this study, the in-hospital mortality was 26.2%. This was consistent with the case fatality rate of 25–28% reported previously. Some studies for a single disease have found that FO has been reported as an independent predictor of death and related to the extension of hospital/ICU stay [6, 16, 19, 24, 25]. Adult studies have found that %FO > 10% is often accompanied by poor clinical prognosis [26]. In children, adverse effects of %FO > 10%, %FO > 15%, and %FO > 20% have been reported. Guidelines for septic shock in children also suggest that %FO > 10% in fluid management can be considered as an indicator for diuretic or RRT and other interventions. According to previous studies, 10% or 20% of early FO may be an important threshold for prognosis. Although there was no significant statistical relationship between %FO > 10% and mortality in our study, %FO > 20% was related to an increase in mortality.

However, the association between early FO and mortality was not found in this study. There was even a weak correlation between early FO and LOS in PICU. Some previous studies also found no significant correlation between FO and mortality [11, 27]. On our multivariate analysis, use of more vasoactive drugs, the presence of MODS, a longer duration of MV, and non-operative reason for PICU admission were positively related to in-hospital mortality. Given the importance of the effects of vasoactive drugs, we also analyzed the number of vasoactive drugs administered. A study of mortality-related factors in CRRT for AKI also confirmed that MV, the use of vasoactive drugs and other factors were related to increased mortality [28]. It is also worth noting that in a study on FO and mortality in 118 children with MV [29], there was a significant correlation between FO and organ dysfunction.

In this study, the PRISM-III score was used as the main marker to evaluate the severity of disease. In children with severe disease, positive fluid balance may be related to more early fluid resuscitation and capillary leakage. Some studies have found that the more serious the disease, the more likely it is to cause an increase in FO. Increase of FO was an independent predictor of adverse effects, and the correlation even remained after excluding the influence of disease severity [20]. Importantly, the adverse effects were mostly concentrated in non-survivors with critical disease; it had less similar conclusions confirmed on surviving patients. In many studies, PRISM score are the main factors used to evaluate disease severity [10, 19, 20, 27, 30–33], but our analysis showed that PRISM-III score was not a risk factor for mortality. Therefore, we propose that PRISM-III score may not indicate severity over the whole course of hospitalization in this study. Many similar situations have been reported previously in the relevant literatures [27, 30].

Sinitsky et al. [11] found that diagnostic category was an independent prognostic factor in a study of the correlation between FO at 48 h and respiratory morbidity. Vidal et al. [14] reported that respiratory and septic shock were related to prolonged MV in a study of the correlation between fluid balance and length of MV in children. In this study, the main reason for admission was even statistically significantly related to mortality. So, the impact of main reason for admission can be better understood. Moreover, We also reported for the first time that the 10% < %FO ≤ 20% group had fewer medical patients and more surgical patients. Another study found that potential etiology and disease severity were independent factors of mortality, and that side effects of FO only occur in the treatment of mild diseases with CRRT [31]. In our study, there were also many prognostic factors with significant differences in different early FO groups.

There still have several reports on multisystem diseases in PICUs [9, 10, 34]. A study on the relationship between mortality and FO in children with severe diseases confirmed that FO was a risk factor for death; however, the correlation analysis was a univariate analysis. Another study in a PICU in South Africa, focusing on FO in children with all severe diseases, showed that high levels of FO were associated with increased mortality. However, it should be noted that the study site and the disease spectrum were different from those of the current study, and most patients did not have high fluid accumulation; %FO > 10% only accounted for 3% of cases. Therefore, it is very important to evaluate the complexity and severity of the diseases, which maybe have a great impact on outcomes.

This study had some shortcomings. 1) This was a retrospective analysis, the information bias was not negligible, and the number of research subjects was small. Although we used the concept of FO in fluid evaluation, the concept of FO was still undefined. 2) Many patients may have had some degree of FO before entering the PICU or before MV, and we could not eliminate its effects. 3) The disease spectrum was complex, and there were defects in the assessment of disease severity.

## Conclusions

In critically ill MV-treated children, owing to the influence of disease severity and intervention measures, the correlations of early FO and %FO > 10% with in-hospital mortality were not clear in this study, but %FO > 20% was related to increased mortality. We suggest that positive FO may have adverse effects. Our study further provides a foundation for the development and evaluation of interventional strategies to mitigate the potential hazards associated with FO. More large prospective pediatric studies are still needed to further explore the threshold of adverse reactions of early FO.

## Data Availability

The datasets used and/or analysed during the current study are available from the corresponding author on reasonable request.

## Abbreviations

AKI: acute kidney injury
ALI: acute lung injury
ARDS: adult respiratory distress syndrome
CIs: confidence intervals
CRRT: continuous renal replacement therapy
FO: fluid overload
IQR: interquartile range
LOS: length of stay
MODS: multiple organ dysfunction syndrome
MV: mechanical ventilation
OR: odds ratio
PICU: pediatric intensive care unit
PRISM-III: third-generation admission pediatric risk of mortality score
SD: standard deviation
